# Out-of-pocket expenditure on community healthcare services at end-of-life among decedents from cardiovascular disease in six European countries and Israel

**DOI:** 10.1186/s13561-023-00449-4

**Published:** 2023-06-13

**Authors:** Aviad Tur-Sinai, Netta Bentur

**Affiliations:** 1 Department of Health Systems Management, The Max Stern Yezreel Valley College, 1930600 Yezreel Valley, Israel; 2 School of Nursing, University of Rochester Medical Center, Rochester, NY 14642-8404 USA; 3 The Minerva Center on Intersectionality in Aging (MCIA), University of Haifa, 3498838 Haifa, Israel; 4 The Stanley Steyer School of Health Professions, Sackler Faculty of Medicine, Tel-Aviv University, Tel-Aviv, Israel

**Keywords:** Cardiovascular disease, Out-of-pocket, Economic capacity, Welfare regime, SHARE, I14, I18, I38, J14

## Abstract

**Objectives:**

Most people who develop chronic diseases, including cardiovascular disease (CVD), live in their homes in the community in their last year of life. Since cost-sharing is common in most countries, including those with universal health insurance, these people incur out of pocket expenditure (OOPE).

The study aims to identify the prevalence and measure the size of OOPE among CVD decedents at end-of-life (EOL) explore differences among countries in OOPE, and examine whether the decedents’ characteristics or their countries’ health policy affects OOPE more.

**Methods:**

SHARE data among people aged 50 + from seven European countries (including Israel) who died from CVD are analyzed. Decedents’ family members are interviewed to learn about OOPE on their relatives’ account.

**Results:**

We identified 1,335 individuals who had died from CVD (average age 80.8 years, 54% men). More than half of CVD-decedent people spend OOPE on community services at EOL and their expenditure varies widely among countries. About one-third of people in France and Spain had OOPE, rising to around two-thirds in Israel and Italy and almost all in Greece. The average OOPE is 391.9 PPT, with wide variance across countries. Significant odds of OOPE exist in the country variable only, and significant differences exist in the amount of OOPE among countries and duration of illness preceding death.

**Conclusions:**

Since improving CVD care efficiency and effectiveness are key aims, healthcare policymakers should broaden the investigation into expanding public funding for community services in order to mitigate OOPE, alleviate the economic burden on households, mitigate forgoing of community services due to price, and reduce rehospitalization.

## Introduction

End-of-life (EOL) care for noncommunicable diseases imposes a substantial economic burden on society, healthcare, and social-care systems as well as patients and their families. Between one-tenth to one-quarter of healthcare expenditure throughout the course of life is concentrated in the last year of life [1, 2].

One of the conditions that have drawn particular attention is cardiovascular diseases (CVD) because its incidence increases with age, driving the growth of healthcare costs. CVD is associated with immense healthcare expenditure [3, 4], estimated at 1%–2% of the healthcare budget in Western countries [5].

Most European countries have introduced different types of universal coverage of a core set of healthcare services [6], with different types of governmental involvement in financing, regulation, and delivery [7]. However, countries differ in the total share of government in healthcare expenditure [8].

Given the need to fund healthcare systems in a way that will guarantee their sustainability and to apply effective cost-containment policies [9], the use of cost-sharing is common in most countries [10]. Charging patients for medical services is seen as a way of shifting costs from the public exchequer to private sources. As a result, additional patient out-of-pocket (OOPE) expenses are prevalent even in countries that have universal healthcare systems or provide health insurance for all. In addition, health-insurance systems and health insurers are increasingly shifting costs of care, especially for medication, to patients by raising deductibles and imposing copayments [11]. However, the level of OOPE spending varies widely among countries, ranging from 2 to 25% of median household income [12].

Acute hospitalization, considered the key driver of CVD costs [13], is almost always included in universal healthcare coverage. However, pursuant to the trend in recent decades of placing community healthcare in the “driver's seat” [14] of the healthcare system, people with chronic diseases, including CVD, live at home in the community during their last months of life, even if they frequently move into and out of acute hospitalization [15, 16]. Moreover, it is well known that most people at end of life prefer to be cared for and die at their home [17]. As a result, they need optimal community medical care and medications, which are not always covered by their health systems or private insurance.

Out-of-pocket expenditure on healthcare is affected not only by country health coverage and regulations but also by patients’ sociodemographic characteristics such as age, gender, educational level, and marital status, as well as socioeconomic-status indicators such as economic capacity and private health insurance [18]. Most studies of OOP costs, however, do not focus on a specific disease [12, 19]. To the best of our knowledge, there is no information on the association between OOP expenditure for CVD decedents and their socio-demographic characteristics. Consequently, three goals are pursued in this study: to measure the proportion of CVD decedents who incurred OOP expenditure in the last twelve months of their lives and the level of the expense; to examine whether there is a difference among countries in OOP spending in the last twelve months of these people's lives; and to examine which of two indicators—differences between countries or in people's characteristics—has a greater impact on this expenditure.

## Methods

### Data source and study sample

The study applies a quantitative approach using the database of the Survey of Health, Aging and Retirement in Europe (SHARE). SHARE-Europe seeks to better understand the dynamics of the growing population of persons aged 50 + and to provide a research infrastructure for public policymaking on behalf of the aging population.

The current study is based on data from Waves 4, 5, 6, and 7 of SHARE, conducted two years apart between 2011 and 2017. After the participants in these waves were located, the data were cross-referenced with a complementary survey conducted under the auspices of SHARE—the SHARE End-of-Life survey—two years or more after the participant was first canvassed. In the complementary survey, family members of deceased persons who had participated in SHARE are approached in order to learn from them about the circumstances of the death, the decedent’s state of health before death, the cause of death, and the expenditure on healthcare in the last year preceding the death. In this manner, information about participants who died between 2011 and 2020 was cross-referenced. The current study focuses on those who died from CVD (heart attack, stroke, or other cardiovascular-related illness such as heart failure, arrhythmia, etc.) in Austria, Germany, France, Spain, Italy, Greece, or Israel.

### Research variables

#### Dependent variables

Family members of CVD-decedents were asked whether their deceased relative had received care from one of the following community outpatient healthcare services: a general practitioner, a specialist practitioner, or medications—in the last twelve months of his or her life. The reason for dealing only with these three outpatient healthcare services is that, as explained above, many people spend the last period before their death at home and often need outpatient medical care and medication. Insofar as the family members answered in the affirmative, they were asked if the decedent had incurred out-of-pocket expenditure, namely, had had to spend money beyond payments received and covered fully by his or her Health Maintenance Organization (HMO) or supplemental health insurance, and how much the person had paid for each of these three services.

By using this information, we first defined the variable of out-of-pocket expenditure for community outpatient healthcare services (a dummy variable: 1 = there was an OOP expenditure for care from a general practitioner, from a specialist practitioner, or on medication; 0 = there was no OOP expenditure for any of these forms of care). The variable was defined in terms of at least one of the types of OOP healthcare expenditure due to the small number of observations in each service. The second dependent variable was the total amount of OOP expenditure on these three services during the person’s last twelve months of life (expressed in PPT, year 2020).

#### Independent variables

The independent variables refer to the former survey, in which the deceased person was interviewed. The independent variables are socio-demographic characteristics (gender, age, living alone, and education) and socioeconomic information, including the financial capacity of the person’s household (a subjective self-assessment relating to the household’s ability to make ends meet: 1. with great difficulty; 2. with some difficulty; 3. fairly easily; 4. easily); having supplemental health insurance (1 = has supplemental health insurance, 0: does not); and whether the person received informal care in the past twelve months (1. Yes; 0. No). In addition, information about the duration of the person’s illness before his or her death was obtained (1. Less than one month; 2. One month or more but less than six months; 3. Six months or more but less than one year; 4. One year or more). Furthermore, a variable was included that represents the first research period (one of two) for which data were collected about every patient. This variable, representing time fixed effects, is dichotomous for each research period, with Wave 4 of SHARE set as the baseline relative to all other periods used in the study.

### Ethics

The SHARE project is operated under the umbrella of the Max Planck Society at the Max Planck Institute for Social Law and Social Policy and is centrally coordinated by the Munich Center for the Economics of Aging. The reseach-ethical assessments of the SHARE project were received from the Ethics Council of the Max Planck Society.

### Statistical analysis

The data were analyzed with the help of STATA Version 15.1. Descriptive analyses of the mean and distribution of the variables were reported for those who had OOP expenditure, as well as for those who had none. Differences between the two groups were assessed using an χ^2^ test for categorical variables and a t-test for continuous variables.

A logistic regression was invoked to identify factors associated with the odds of having OOP expenditure on healthcare services among patients who died from a cardiovascular disease. Adjusted odds ratios with corresponding 95% confidence intervals were calculated. A linear regression was invoked to identify factors associated with OOP expenditure on healthcare services. The level of significance was accepted as α = 0.05.

## Results

We identified 1,335 individuals aged 50 + who had died from CVD: 190 individuals in 2011, 328 individuals in 2013, 457 individuals in 2015 and 360 individuals in 2017. Their average age was 80.8 years (S.D. = 9.1 years). Some 54 percent were men, they had eight years of schooling on average, and 27. 9 had supplemental health insurance, with no difference between those who had incurred OOPE and those who had not. Out of the total population, 20.5 percent had dire financial difficulties, 57.5 had some difficulties, and 22.0 percent had no difficulties.

Of the total population, 741 (55.7 percent) spent out of pocket for community healthcare services (general-practitioner care, specialist-practitioner care, or medications) in the last twelve months of their lives. Among those with OOPE, 50.2 percent live alone as against 44.6 percent who live alone among those with no OOPE. In addition, among those who incurred OOPE, 25.3 percent encountered great financial difficulties as against 14.1 percent of those who had not incurred such expenditure. Furthermore, 20.5 percent of those with OOPE, as against 24.0 percent of those who had not incurred such expenditure, reported having no financial difficulties. In addition, 14.7 percent of those who incurred OOPE had been ill for 1–6 months before dying, whereas 18.1 percent of those who had had no such expenditure had been ill for this length of time before passing. Furthermore, 40.1 percent of those incurring OOPE had been ill for more than one year before dying as against 32.9 percent of those who had not had OOPE before passing (Table 1). A similar statistical descriptive for each country appears Table 4 in Appendix.

**Table 1:** Characteristics of CVD decedents (percent)

		Had no OOPE (*N* = 594, 44.49%)	Had OOPE (*N* = 741, 55.51%)	F/X^2^	ALL (*N* = 1335)
Gender	Female	46.30	46.56	0.01	46.44
	Male	53.70	53.44		53.56
Age (mean and SD)		80.45 (9.67)	81.04 (8.67)	1.39	80.78 (9.13)
Living alone	No	55.39	49.80	4.13*	52.28
	Yes	44.61	50.20		47.72
Education (mean and SD)		7.82 (4.83)	7.68 (4.79)	0.29	7.74 (4.81)
Economic capacity (household’s ability to make ends meet)	With great difficulty	14.10	25.31	27.09***	20.49
	With some difficulty	29.49	29.32		29.39
	Fairly easily	32.42	24.90		28.13
	Easily	23.99	20.47		21.99
Health insurance	No insurance	73.65	70.83	1.30	72.08
	Has supplemental health-insurance coverage	26.35	29.17		27.92
Informal caregiving	No	5.50	4.26	1.09	4.81
	Yes	94.50	95.74		95.19
How long ill before death	Less than 1 month	39.36	38.04	9.36*	38.63
	1–6 months	18.07	14.67		16.19
	6–12 months	9.63	7.20		8.28
	1 year or more	32.94	40.08		36.90

^*^P < 0.05, **P < 0.01, ***P < 0.001

**Fig. 1:**
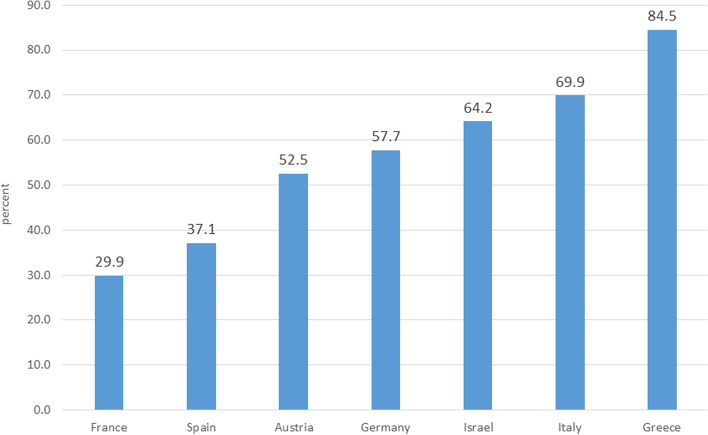
Probability of out-of-pocket expenditure on community healthcare services among CVD decedents in the last 12 months of life (percent)

Differences were found among countries in paying out of pocket for community healthcare. About one third of respondents in France (29.9%) and 37.1% in Spain reported OOPE, rising to between one-half and two-thirds in Austria (52.5%), Germany (57.7%), Israel (64.4%), and Italy (69.9%). Almost all decedents in Greece (84.5%) spent out of pocket on outpatient medical care in the last year of their lives (Fig. 1).

**Fig. 2:**
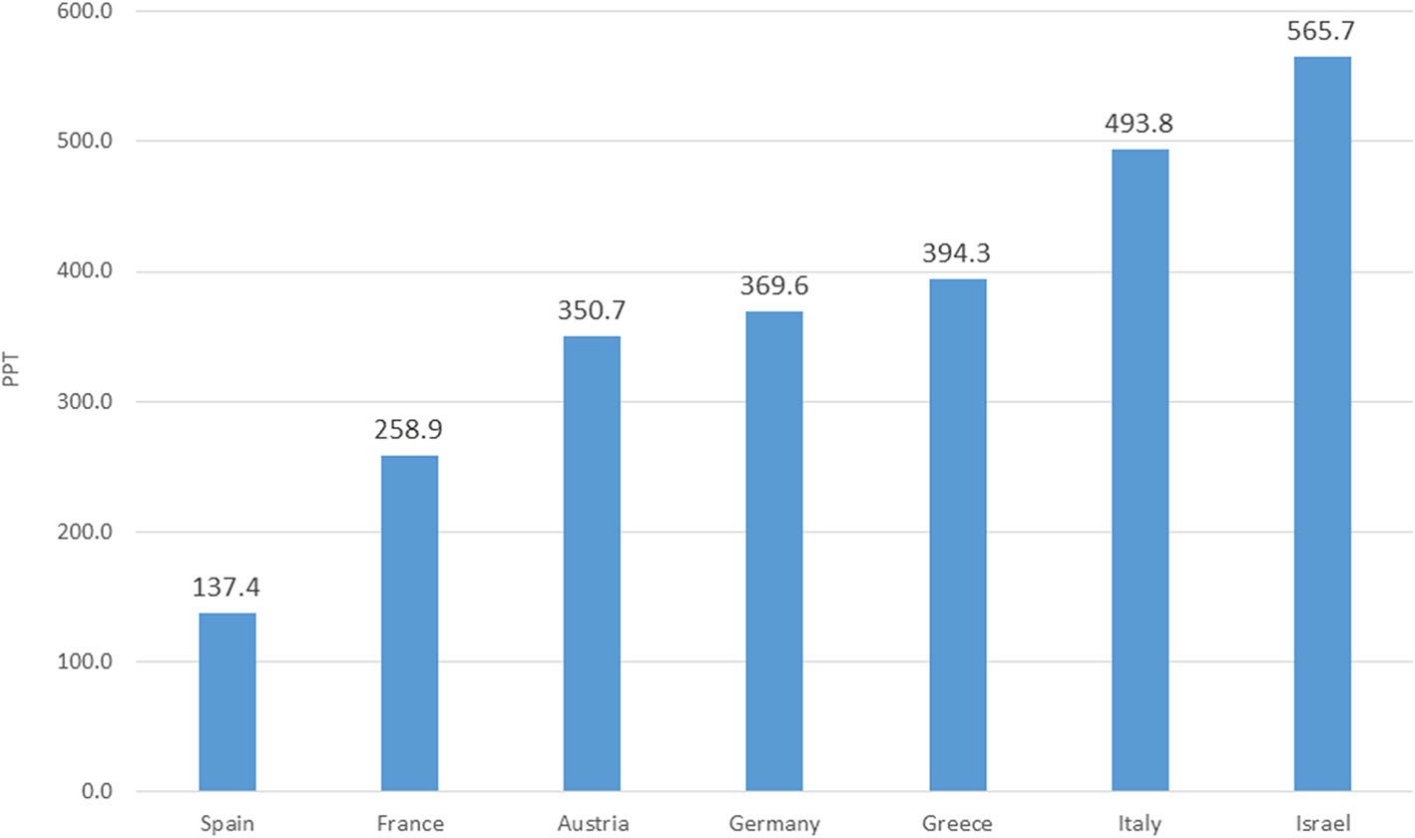
Total out-of-pocket expenditure on community healthcare services among CVD decedents in the last 12 months of life (expressed in Purchasing Power Parity)

The average OOPE in the last twelve years of life of a person who died from CVD was 391.9 PPT (S.D 459.1 PPT). The average OOPE was 137.4 PPT and 258.9 PPT in Spain and France, respectively. In Italy and Israel, in contrast, it was more than twice as high, at 493.8 PPT and 565.7 PPT, respectively (Fig. 2).

The odds of spending out of pocket for community healthcare services appear in Table 2. In the first model, the odds were estimated against the patients’ characteristics. The probability of OOPE was found higher among single households (OR = 1.463, 95% CI = 1.124–1.905, *P* < 0.01) and lower in tandem with ease in economic capacity (OR = 0.787, 95% CI = 0.701–0.882, *P* < 0.001) (Model 1). In the second model, the odds were estimated against the dichotomous variables that represent the countries investigated (with France as the reference country). No difference was found in the odds ratio between France and Spain. In contrast, much variance appeared in the odds ratios of the occurrence of OOP expenditure on outpatient healthcare services in all other countries (Model 2). In the third model, the odds were estimated against patients’ characteristics and countries. Only the single (OR = 1.429, 95% CI = 1.080–1.890, *P* < 0.05) and the country variables showed a significant relation with the odds of spending out of pocket. The likelihood of having OOPE is 350.4 percent greater in Austria than in France or Spain (OR = 3.504, 95% CI = 1.797–6.832, *P* < 0.001), whereas the odds of OOPE are 1053.9 percent higher in Greece than in France or Spain (OR = 11.539, 95% CI = 6.244–21.323, *p* < 0.001). Time fixed effects were included in each of the estimations; they were not found significant. (The findings are not presented).

**Table 2:** Probability of out-of-pocket expenditure on community healthcare services among CDV decedents (odds ratios) (95% CI) (Logit model)

	Model 1		Model 2		Model 3	
	OR	95% CI	OR	95% CI	OR	95% CI
Male	1.072 (0.14)	(0.827, 1.390)			1.050 (0.15)	(0.797, 1.383)
Age	1.008 (0.01)	(0.995, 1.022)			1.006 (0.01)	(0.991, 1.021)
Living alone	1.463** (0.20)	(1.124, 1.905)			1.429* (0.20)	(1.080, 1.890)
Education	1.008 (0.01)	(0.983, 1.034)			0.994 (0.01)	(0.966, 1.1.023)
Economic capacity (1 = with great difficulty, 4 = easily)	0.787*** (0.05)	(0.701, 0.882)			1.003 (0.07)	(0.877, 1.149)
Health insurance (1 = yes, 0 = no)	0.961 (0.03)	(0.901, 1.025)			0.986 (0.04)	(0.915, 1.1.063)
How long ill before death	1.077 (0.05)	(0.987, 1.175)			1.090 (0.05)	(0.990, 1.199)
Informal caregiving (1 = yes, 0 = no)	1.227 (0.34)	(0.717, 2.101)			1.729 (0.64)	(0.836, 3.577)
Austria			2.589*** (0.66)	(1.566, 4.282)	3.504*** (1.19)	(1.797, 6.832)
Germany			3.195*** (0.90)	(1.834, 5.565)	3.528*** (1.09)	(1.929, 6.450)
Spain			1.383 (0.31)	(0.885, 2.160)	1.277 (0.32)	(0.781, 2.088)
Italy			5.446*** (1.33)	(3.368, 8.805)	5.2000*** (1.41)	(3.061, 8.838)
France			Ref		Ref	
Greece			12.772*** (3.59)	(7.364, 22.153)	11.539*** (3.62)	(6.244, 21.323)
Israel			4.195*** (1.16)	(2.435, 7.227)	3.757*** (1.18)	(2.028, 6.962)
Constant	0.769 (0.51)	(0.210,2.806)	0.427*** (0.09)	(0.287, 0.634)	0.124** (0.10)	(0.027, 0.578)
N	1232		1335		1232	
Log-likelihood	-823.943		-823.842		-747.341	

^*^P < 0.05, **P < 0.01, ***P < 0.001

Estimation of the odds of OOPE on community healthcare services in each country shows that the socio-demographic and economic variables are not significant in any country except Italy, where the better-off a household is, the lower are the odds of its incurring OOPE, and Greece, where the probability of OOP expenditure rises in tandem with individuals’ age (without table).

Estimating the determinants of the amount of OOPE on community healthcare services, it was found that OOPE falls in opposition to the ability of the person’s household to make ends meet. Similarly, OOPE was found positively correlated with the duration of a person’s illness before death (Table 3, Model 1). In the second model, OOPE levels in France and Spain are not significantly different from each other, whereas a significant difference between France and all other countries appeared (Model 2). In the third model, it was found that OOPE levels in France and Spain were not significantly different from each other, such expenditure in all other countries was significantly higher than that in France and OOP expenditure in Israel, and OOPE in Greece and Italy was the highest relative to France (Model 3). Two patient-related characteristics also explain OOPE: duration of illness before death (positive explanatory power) and country of residence. Time fixed effects were included in each of the estimations; they were not found significant. (The findings are not presented).

**Table 3:** Out-of-pocket expenditure on community healthcare services among CVD decedents, OLS regression model (dependent variable: ln (out-of-pocket expenditure)

	Model 1		Model 2		Model 3	
	Coef	95% CI	Coef	95% CI	Coef	95% CI
Male	-0.001 (0.10)	(-0.196, 0.195)			-0.025 (0.09)	(-0.198, 0.147)
Age	0.006 (0.01)	(-0.005, 0.016)			0.007 (0.00)	(-0.003, 0.016)
Living alone	0.013	(-0.184, 0.210)			0.021	(-0.152, 0.194)
	(0.10)				(0.09)	
Education	0.005 (0.01)	(-0.014, 0.024)			-0.003 (0.01)	(-0.021, 0.015)
Economic capacity (1 = with great difficulty, 4 = easily)	-0.124** (0.04)	(-0.208, -0.040)			0.069 (0.04)	(-0.015, 0.153)
Health insurance (1 = yes, 0 = no)	-0.012 (0.02)	(-0.058, 0.035)			0.014 (0.02)	(-0.031, 0.059)
How long ill before death	0.140*** (0.03)	(0.076, 0.204)			0.156*** (0.03)	(0.099, 0.213)
Informal caregiving (1 = yes, 0 = no)	-0.381 (0.22)	(-0.816, 0.054)			-0.293 (0.25)	(-0.779, 0.192)
Austria			0.956*** (0.21)	(0.544, 1.368)	1.006*** (0.24)	(0.532, 1.480)
Germany			1.029*** (0.22)	(0.593, 1.464)	1.057*** (0.23)	(0.611, 1.502)
Spain			0.175 (0.20)	(-0.211, 0.561)	0.183 (0.20)	(-0.215, 0.581)
Italy			1.543*** (0.19)	(1.164, 1.923)	1.656*** (0.20)	(1.262, 2.049)
France			Ref		Ref	
Greece			1.414*** (0.19)	(1.034, 1.795)	1.560*** (0.20)	(1.158, 1.962)
Israel			1.213*** (0.21)	(0.795, 1.630)	1.232*** (0.22)	(0.792, 1.672)
Constant	5.161*** (0.52)	(4.133, 6.194)	4.268*** (0.18)	(3.922, 4.614)	3.362*** (0.53)	(2.323, 4.401)
N	701		741		701	
Adj. R^2^	0.0301		0.2061		0.2526	

^*^P < 0.05, **P < 0.01, ***P < 0.001

Estimation of total OOPE in each country shows that the socio-demographic and economic variables are not significant in any country except Germany, where people who have supplemental health insurance spend more out-of-pocket than do those who lack such coverage (without table).

## Discussion

It was found in this study that more than half of CVD decedents spent out of pocket on community services in their last year of life. In addition, large differences were found among countries regarding OOP spending and the country variable accounts for nearly all of the impact on the odds of out-of-pocket expenditure (OOPE) and the extent of its level.

The share of OOPE expenditure in the last twelve months of life among people who died from CVD is highest in Israel, Italy, and Greece, and lowest in France, Spain, Austria, and Germany. When the extent of OOPE is measured, the same tendency recurs.

The inquiry in this article related to OOPE on healthcare services in the community and not to total healthcare outlays. However, it seems possible to draw inferences from this investigation, with due caution, about financial comportment in the various countries.

Per-capita GDP—an indicator that represents the economic resources on which a country may call for the delivery of public services to its inhabitants, proxies a government’s ability to provide its residents with healthcare services—is contingent both upon the economic resources available to it and upon its perception of socio-public policy. Our comparison of the countries investigated shows that the countries are strongly differentiated by the economic resources that they can pledge to public services for their residents. Whereas per-capita GDP is highest in Austria, Germany, and France, at 55,614 PPT, 52,548 PPT, and 45,322 PPT in 2020, respectively, it is not much more than half of that in Greece (28,662 PPT). In Italy, Spain, and Israel, per-capita GDP is 25–30 percent lower than in Austria [8]. This variance in the economic resources that can be used to deliver economic welfare services to residents may explain the ability of a given country to fund healthcare services.

The order of the countries in relation to the findings of the study is also inverse to the share of government healthcare expenditure in total healthcare expenditure. Greece and Israel had the lowest proportions of government in total healthcare spending, at 61.4 percent and 63.3 percent, respectively; France and Germany had the highest rates, at 78.0 percent and 83.9 percent [8].

Esping-Andersen’s welfare-state model [20] may offer an explanation of these findings. In this model, countries that apply a continental welfare regime subject assistance to means testing and offer modest social-insurance plans that typically target lower-income individuals; such is the case in Germany, Austria, and France. In contrast, in countries that have a Mediterranean welfare regime, citizens receive public assistance only when personal resources are exhausted and traditional values encourage families to help out (e.g., Italy, Spain, Greece, and Israel).

Therefore, in countries that invoke the continental welfare regime, one expects to find generous allocation of public resources for the funding of residents’ healthcare and social services; such a paradigm is expected to lower the odds of OOPE by residents and alleviate their financial burden in funding healthcare services. In countries that use the Mediterranean welfare regime, in contrast, parsimonious diversion of public resources to the funding of residents’ healthcare and social expenditure is expected; this is likely to increase the odds of OOP spending and the economic burden that residents bear in funding their healthcare services.

Regarding the relation between the probability of OOP and patients’ characteristics, it was lower among those with better economic capacity than among others. When countries were entered into the analysis, however, only the country variable was found to have a significant association with the odds of OOPE. As for the amount of OOPE among those who incurred it, an association was found only with lengthier duration of illness before death and the country variable. Gender, age, education, economic capacity, having supplemental health insurance, and having informal caregiving were not found to be related to OOP expenditure on community services.

Although no studies on the association between OOPE and the socioeconomic status of CVD decedents were found, it is important to note that many studies point to a connection between CVD and epidemiological indicators such as mortality, recurrent morbidity, and receipt of care, that may shed light on the importance of these characteristics in the context of the patients. Studies show that lower socioeconomic status is inversely associated with higher risks of CVD diseases, recurrent CVD disease, and mortality [21, 22]. Among one-year survivors of a first myocardial infarction, for example, recurrence was predicted by unstable income, level of education, and marital status [23]. In addition, low socioeconomic status was found to be associated with suboptimal medical care, less access to treatment [24], poorer treatment adherence [25, 26], and lower secondary prevention and effectiveness of intervention [27]. However, the discussion of the association between socioeconomic status and health-system coverage on the quality of community care for CVD is inconclusive. Regarding pharmacological treatment, several studies found that access to medication was lower among persons of low socioeconomic status than among those of high status [28, 29]; other studies, however, found no association between socioeconomic status and access to pharmacological care [30].

It may be possible that in addition to the patients’ socioeconomic and demographic characteristics that were included in this study, lifestyle, social determinants of health, cultural, ethnic, and sociological characteristics may explain variance among patients more than would the traditional characteristics included in this study [31].

Our findings have significant implications for healthcare policymakers. They highlight the need for reconsideration of resources allocation for community care following CVD. Since much of the national economic burden of CVD relates to hospitalization and rehospitalization due to obstacles to optimal community services, lowering these obstacles may improve the quality of care, reduce readmission, and, in turn, alleviate the overall financial burden of CVD [27]. Studies show that despite the high risk of readmission among patients hospitalized for CVD, most patients do not visit a physician within a week of discharge and those who have higher early follow-up rates have a lower risk of readmission within a month [15]. However, follow-up community care faces barriers such as patients’ advanced age, high rates of comorbidity and complex medical treatment, poor access to specialists in the community, and cost, including OOP payments for physicians and medication [32, 33]. Thus, if governments lower these barriers, they may reduce rehospitalization following CVD. OOPE on visits to specialist cardiologists is of special importance because patients discharged from hospitals who have the highest rates of early follow-up by a cardiologist are at less risk of mortality [15, 34]. A better understanding of the exact components of OOPE is needed, and the inclusion of those with special benefits in universal healthcare coverage should be considered.

This study has several limitations. First, the data are based on self-estimation of OOPE on community healthcare, raising the possibility of under- or over-estimate. Second, the findings are based on proxy respondents of the deceased patients, possibly resulting in a memory problem that may create recall and social-desirability bias. However, interviewing bereaved families in order to explore care up to end of life is common in research [35] and studies have shown that the use of proxy respondents is especially appropriate when some objective measure of costs, such as bills, reduces recall bias [36]. Third, the respondents were sampled from private households, meaning that people who died in institutions were not included. Fourth, the small sample size in each country may have affected the proportions. Fifth, we studied all CVD types together due to the sample-size limitation, but different types of CVD may cause different degrees of OOPE. Finally, other health conditions and comorbidities that may affect OOP spending were not included in the study. To reveal and understand the feelings, opinions, and needs of patients and their caregivers, shedding light on their diverse problems, especially financial ones, follow-up qualitative research is recommended.

## Conclusions

Improving the efficiency and effectiveness of CVD care is a matter of key focus among healthcare policymakers in reducing CVD-related costs, morbidity, and mortality. Therefore, an understanding of the end-of-life care of CVD patients is crucial for the development of policies that will address the challenges of providing this population with optimal care.

Our study revealed that the welfare regime to which the individual belongs is found dominant in explaining the generosity of public investment in the healthcare system and in easing the financial burden on households for funding healthcare services. Accordingly, the expansion of public funding of CVD community care may lower their out-of-pocket expenditure, mitigate non-use of community services due to cost, and reduce hospital readmissions. This is important in all countries included in the study and especially so in those where OOPE is highest.

## Data Availability

The datasets analysed during the current study are available in the Survey of Health Ageing and Retirement in Europe (SHARE) repository, http://www.share-project.org/home0.html.

## Appendix

**Table 4:** Characteristics of CVD decedents, by country (percent)

a. Austria						
			Had no OOPE (*N* = 76, 47.5%)	Had OOPE (*N* = 84, 52.5%)		F/X^2^
Gender	Female		51.32	53.57		0.08
	Male		48.68	46.43		
Age (mean and SD)			78.28 (10.76)	79.25 (9.09)		0.38
Living aloneNo			34.21	52.38		5.35*
Yes			65.79	47.62		
Education (mean and SD)			8.39 (4.63)	8.68 (4.50)		0.15
Economic capacity (household’s ability to make ends meet)	With great difficulty		0	4.94		15.80***
	With some difficulty		30.77	7.41		
	Fairly easily		35.38	44.44		
	Easily		33.85	43.21		
Health insurance	No insurance		78.95	78.57		0.00
	Has supplemental health-insurance coverage		21.05	21.43		
**P* < 0.05, ***P* < 0.01, ****P* < 0.001						
b. Germany						
			Had no OOPE (*N* = 44, 42.31%)	Had OOPE (*N* = 60, 57.69%)		F/X^2^
Gender		Female	45.45	28.33		3.25
		Male	54.55	71.67		
Age (mean and SD)			75.93 (9.09)	77.02 (10.39)		0.31
Living aloneNo			68.18	66.67		0.27
Yes			31.82	33.33		
Education (mean and SD)			11.55 (4.25)	11.77 (3.81)		0.08
Economic capacity (household’s ability to make ends meet)		With great difficulty	7.89	1.75		4.42
		With some difficulty	15.79	7.02		
		Fairly easily	36.84	40.35		
		Easily	39.47	50.88		
Health insurance		No insurance	88.64	79.66		1.47
		Has supplemental health-insurance coverage	11.36	20.34		
**P* < 0.05, ***P* < 0.01, ****P* < 0.001						
c. Spain						
			Had no OOPE (*N* = 244, 62.89%)	Had OOPE (*N* = 144, 37.11%)		F/X^2^
Gender		Female	43.85	47.22		0.42
		Male	56.15	52.78		
Age (mean and SD)			81.45 (8.74)	82.76 (8.35)		2.10
Living aloneNo			60.66	43.75		10.43***
Yes			39.34	56.25		
Education (mean and SD)			6.48 (5.01)	5.92 (4.78)		1.20
Economic capacity (household’s ability to make ends meet)		With great difficulty	14.72	13.24		1.15
		With some difficulty	31.17	30.88		
		Fairly easily	35.06	32.35		
		Easily	19.05	23.53		
Health insurance		No insurance	76.13	80.56		1.02
		Has supplemental health-insurance coverage	23.87	19.44		
**P* < 0.05, ***P* < 0.01, ****P* < 0.001						
d. Italy						
			Had no OOPE (*N* = 74, 30.09%)	Had OOPE (*N* = 172, 69.91%)		F/X^2^
Gender	Female		43.24	45.93		0.15
	Male		56.76	54.07		
Age (mean and SD)			81.08 (9.45)	80.09 (8.81)		0.63
Living aloneNo			59.46	51.16		1.43
Yes			40.54	48.84		
Education (mean and SD)			6.36 (4.59)	6.24 (4.42)		0.04
Economic capacity (household’s ability to make ends meet)	With great difficulty		15.49	31.98		10.65*
	With some difficulty		36.62 29.58	38.95 19.77		
	Fairly easily		18.31	9.30		
	Easily					
Health insurance	No insurance		81.08	77.19		0.46
	Has supplemental health-insurance coverage		18.92	22.81		
**P* < 0.05, ***P* < 0.01, ****P* < 0.001						
e. France						
			Had no OOPE (*N* = 82, 70.09%)	Had OOPE (*N* = 35, 29.91%)		F/X^2^
Gender	Female		52.44	51.43		0.01
	Male		47.56	48.57		
Age (mean and SD)			80.73 (11.45)	80.46 (9.83)		0.02
Living aloneNo			50.00	48.57		0.02
Yes			50.00	51.43		
Education (mean and SD)			9.18 (2.89)	9.91 (3.76)		1.31
Economic capacity (household’s ability to make ends meet)	With great difficulty		1.75 17.39	15.63 21.88		10.46*
	With some difficulty		43.48	21.88		
	Fairly easily		37.68	40.63		
	Easily					
Health insurance	No insurance		75.31	80.00		0.30
	Has supplemental health-insurance coverage		24.69	20.00		
**P* < 0.05, ***P* < 0.01, ****P* < 0.001						
f. Greece						
			Had no OOPE (*N* = 31, 15.50%)	Had OOPE (*N* = 169, 84.50%)	F/X^2^	
Gender	Female		38.71	50.89	1.55	
	Male		61.29	49.11		
Age (mean and SD)			78.26 (9.94)	83.39 (6.75)	12.87**	
Living aloneNo			64.52	42.60	5.06*	
Yes			35.48	57.40		
Education (mean and SD)			7.13 (4.22)	6.91 (3.88)	0.08	
Economic capacity (household’s ability to make ends meet)	With great difficulty		54.84 38.71	52.07 34.91	1.35	
	With some difficulty		3.23	9.47		
	Fairly easily		3.23	3.55		
	Easily					
Health insurance	No insurance		74.19	71.60	0.09	
	Has supplemental health-insurance coverage		25.81	28.40		
**P* < 0.05, ***P* < 0.01, ****P* < 0.001						
g. Israel						
			Had no OOPE (*N* = 43, 35.83%)	Had OOPE (*N* = 77, 64.17%)	F/X^2^	
Gender	Female		51.16	41.56	1.03	
	Male		48.84	58.44		
Age (mean and SD)			83.19 (7.85)	80.17 (8.45)	3.70	
Living aloneNo			46.51	58.44	1.58	
Yes			53.49	41.56		
Education (mean and SD)			11.02 (4.33)	10.60 (5.20)	0.21	
Economic capacity (household’s ability to make ends meet)	With great difficulty		26.83 31.71	15.79 35.53	2.80	
	With some difficulty		17.07	26.32		
	Fairly easily		24.39	22.37		
	Easily					
Health insurance	No insurance		18.60	16.00	0.14	
	Has supplemental health-insurance coverage		81.40	84.00		
**P* < 0.05, ***P* < 0.01, ****P* < 0.001						
